# Preclinical Molecular Imaging for Precision Medicine in Breast Cancer Mouse Models

**DOI:** 10.1155/2019/8946729

**Published:** 2019-09-22

**Authors:** M. F. Fiordelisi, L. Auletta, L. Meomartino, L. Basso, G. Fatone, M. Salvatore, M. Mancini, A. Greco

**Affiliations:** ^1^IRCCS SDN, Napoli, Italy; ^2^Centro Interdipartimentale di Radiologia Veterinaria, Università degli Studi di Napoli Federico II, Naples, Italy; ^3^Dipartimento di Medicina Veterinaria e Produzioni animali, Università degli Studi di Napoli Federico II, Naples, Italy; ^4^IBB CNR, Napoli, Italy; ^5^Dipartimento di Scienze Biomediche Avanzate, Università degli Studi di Napoli Federico II, Naples, Italy

## Abstract

Precision and personalized medicine is gaining importance in modern clinical medicine, as it aims to improve diagnostic precision and to reduce consequent therapeutic failures. In this regard, prior to use in human trials, animal models can help evaluate novel imaging approaches and therapeutic strategies and can help discover new biomarkers. Breast cancer is the most common malignancy in women worldwide, accounting for 25% of cases of all cancers and is responsible for approximately 500,000 deaths per year. Thus, it is important to identify accurate biomarkers for precise stratification of affected patients and for early detection of responsiveness to the selected therapeutic protocol. This review aims to summarize the latest advancements in preclinical molecular imaging in breast cancer mouse models. Positron emission tomography (PET) imaging remains one of the most common preclinical techniques used to evaluate biomarker expression *in vivo*, whereas magnetic resonance imaging (MRI), particularly diffusion-weighted (DW) sequences, has been demonstrated as capable of distinguishing responders from nonresponders for both conventional and innovative chemo- and immune-therapies with high sensitivity and in a noninvasive manner. The ability to customize therapies is desirable, as this will enable early detection of diseases and tailoring of treatments to individual patient profiles. Animal models remain irreplaceable in the effort to understand the molecular mechanisms and patterns of oncologic diseases.

## 1. Introduction

Precision or personalized medicine is becoming increasingly important in the fields of biomedical and clinical research. These two terms, personalized medicine and precision medicine, have been used interchangeably, although they describe different aspects of a common problem. Nonetheless, the shared aim of both personalized and precision medicine is to obtain an early and accurate diagnosis, predict disease evolution and therapy response, and reduce occurrence of therapeutic failures [1–5].

Preclinical imaging of animal models represents an invaluable tool in studying the etiopathogenesis of and therapeutic responses in various human pathologies [6]. In particular, molecular imaging techniques are important because they can be used to assess biological processes at the cellular and molecular levels, enabling detection of disease in very early or presymptomatic stages, and to estimate the efficacy of novel therapies, such as personalized, targeted, and combinational therapies [2, 7, 8]. The ability to study the same animal model of human disease using different techniques, i.e., with a multimodal approach, constitutes an additional advantage for both diagnosis and therapy. Preclinical imaging allows longitudinal studies to be conducted noninvasively and in real time. All these features lead to a reduction in the number of animals required for experimentation, as well as in the cost of biomedical research and drug development, while providing statistically relevant results [9, 10]. Preclinical molecular imaging has been applied for neurological, cardiovascular, and oncologic diseases; however, it particularly has great potential for use in personalized cancer therapy, enabling the study of biological processes and therapy response in individual patients [11]. The assessment of biological properties of tumors, such as metabolism, proliferation, hypoxia, angiogenesis, apoptosis, and gene and receptor expression, contributes to the realization of precision medicine [12, 13], owing to the possibility of monitoring physio-pathological processes *in vivo*, detecting therapeutic responses, identifying nonresponders at an early stage, and enabling the switch to novel therapeutic approaches [14, 15].

This review aims to report the latest advances in precision medicine obtained with the application of molecular imaging techniques in mouse models of breast cancer. The techniques, tracers, and models are summarized in Table 1.

## 2. Breast Cancer Mouse Models

The most common oncologic animal models, i.e., those utilizing a xenograft, are based on the subcutaneous injection of human-derived cell lines in immunodeficient mice. However, these models are not always representative of the overall heterogeneity detectable in naturally occurring cancers and may thus lack predictive value [26]. Hence, models that more closely mirror the heterogeneity of human tumors are necessary for more efficient drug development, i.e., the use of transgenic or patient-derived tumor xenograft (PDX) models [14, 27–30]. These models summarize the biological characteristics of the original disease, thus having much stronger predictive value for the clinical outcome [26, 31]. In any case, mouse models allow the mechanisms of drug resistance to be studied, enabling patient stratification and assignment of nonresponders to novel, and potentially more effective, therapies [26, 31].

### 2.1. Breast Cancer Subcategories

Personalized medicine for breast cancer could be considerably advantageous in terms of healthcare and socioeconomic impact because breast cancer is the second most diffuse as well as the second most common oncologic cause of death [15]. In precision medicine for breast cancer, the peculiar molecular characteristics of different cancer subtypes may help the stratification of patients, as well as the development and evolution of novel therapeutic strategies. Molecular subtypes can be distinct, for example, on the basis of their hormone receptor status. Luminal breast cancers are typically hormone receptor-positive, whereas tumors expressing the human epidermal growth factor receptor 2 (HER2) are usually hormone receptor-negative [14, 15]. The third main subtype, triple-negative breast cancer (TNBC), represents a diverse group of tumors and is characterized by the absence of estrogen, progesterone, and HER2 receptors. Specific interventions for this type of tumor are difficult owing to a high level of heterogeneity within this subtype and owing to the absence of well-defined molecular targets [29, 32].

The HER2, also called the avian erythroblastosis oncogene B (ErbB2), is a transmembrane receptor, which is included in the epidermal growth factor receptor family of tyrosine kinases. It predefines pathways that promote various cellular processes, including proliferation, differentiation, angiogenesis, and antiapoptotic functions [18]. The HER2 is associated with high-grade breast tumors, and its overexpression is considered a marker of aggressiveness and malignancy, as well as an index of resistance to conventional chemotherapy. Analysis of HER2 expression is important for monitoring treatment response and efficacy, particularly with regard to trastuzumab, a humanized monoclonal antibody that specifically binds to the HER2, inhibiting the growth of tumor cells and decreasing HER2 expression [23, 33, 34].

Estrogen receptor-*α* (ER*α*) and progesterone receptor (PR) are expressed in most human breast cancers and are important therapeutic targets. Hence, there is a need to identify ER*α*-positive (ER*α*+)/PR-positive (PR+) tumors, which will likely respond to specific hormonal therapy [25].

Besides these main subcategories, with respect to precision medicine, other receptors expressed in tumor tissues in general, as well as specifically in breast cancer, should be considered. Among such receptors, the vascular endothelial growth factor receptor 2 (VEGFR2) has been used as a target for precision diagnostic imaging as well for precision therapy. The VEGFR2 is highly expressed during early tumor development, and its expression is linked to the onset of neoangiogenesis [35].

## 3. Positron Emission Tomography

Positron emission tomography (PET) is a nuclear medicine imaging technique, which can be used to investigate metabolic processes in the body. Positron-emitting (*ß*+) isotopes can be linked to various substances, e.g., 18 fluorine (^18^F) to 2-deoxy-2-(^18^F)-fluoro-d-glucose (FDG) for glucose metabolism, and their solutions are injected intravenously in patients as well as in animals prior to image acquisition at specific time points or dynamically over time. The system detects pairs of photons in the gamma-ray spectrum of 511 keV, which are produced by the annihilation reaction between *ß*+ emitted by the radioisotope and electrons present in the surrounding medium. Different types of radioactive tracers exist, and their selection depends on the pathology/metabolic process being studied. The aforementioned radiotracer, ^18^F-FDG, which measures glucose metabolism, is the most commonly used radiopharmaceutical in oncology. However, other radiotracers are used to quantify other cellular processes, for example, cell proliferation can be measured with ^18^F-fluoro-3′-deoxy-3′-L-fluorothymidine (^18^F-FLT), which is a substrate of the thymidine-kinase-1 during the S-phase of mitosis [36], or ^18^F-fluoro-misonidazole (18F-FMISO), which is a specific radiotracer to study hypoxia in the tumor microenvironment [37]. Moreover, positron-emitting isotopes can be used for biodistribution studies of novel drugs [38].

### 3.1. Precision Imaging

The possibility of *in vivo* quantification of HER2 receptors was assessed using preclinical PET imaging with 3.7–4.4 MBq of N-2-(4-^18^F-fluorobenzamido)ethylmaleimide–^18^F-FBEM-Z_HER2:342_-Affibody molecule. The tracer was administered to female athymic nude mice bearing xenografts from human breast cancer cell lines MDA-MB-361, MCF7, MDA-MB-468, MCF7/clone18, or BT474. These cell lines showed five different levels of HER2 expression, as demonstrated *ex vivo* by immunohistochemistry: (1) BT474, very high; (2) MCF7/clone18, high; (3) MDA-MB-361, medium; (4) MCF7, very low, and (5) MDA-MB-468, negative. The results showed that ^18^F-FBEM-Z_HER2:342_-Affibody rapidly accumulated in HER2-positive tumors and was just as rapidly eliminated from the blood and normal tissues. Indeed, significant differences in the uptake of the radiolabeled affibody were recorded between tumor and normal tissues and among different breast cancer cell lines (BT474 and MCF7/clone18 showed high uptake, MCF7 and MDA-MB-361 showed a very low uptake, and MDA-MB-468 tumors showed no uptake). These results suggest that the ^18^F-FBEM-Z_HER2:342_-Affibody molecule can be used to quantify HER2 expression *in vivo* [16, 17].

### 3.2. Therapy Response and Detection of Responders vs. Nonresponders

17-(Dimethylaminoethylamino)-17-demethoxygeldanamycin (17-DMAG) is an inhibitor of heat shock protein (Hsp) 90, which is known to decrease HER2 expression. The PET acquisitions with ^18^F-FBEM-Z_HER2:342_-Affibody were performed before and after treatment with four doses of 17-DMAG. The effect of the 17-DMAG treatment on HER2 expression was compared between mice bearing BT474 and MCF7/clone18, and a lower level was found in MCF7/clone18. These results suggest that ^18^F-FBEM-Z_HER2:342_-Affibody can be used not only to quantify the HER2 expression *in vivo* but also to monitor its variations in response to therapeutic interventions [16, 17].

Similarly, the HER2 expression levels were evaluated in breast xenografts mouse models, in response to trastuzumab. For PET scans, animals were injected with 3.7–6.7 MBq of ^18^F-FBEM-HER_2:342_-Affibody via the lateral tail vein and were scanned before the treatment, at 48 h and 2 weeks after the beginning of therapy. At each time point, the tracer uptake in the tumor lesion was quantified and the results were normalized to baseline. The analysis indicated a clear decrease in radiotracer uptake as soon as after the first administration of trastuzumab in the treated mice compared to controls, most likely as a result of the reduction in HER2 levels. The reduction in ^18^F-FBEM-HER_2:342_-Affibody uptake was thus considered a proof of the antitumor activity of trastuzumab. However, there were differences in the radiotracer uptake at the end of the treatment responses, probably due to a heterogeneous response to the lower dosage. These findings were confirmed by immunohistochemical analysis, which showed a high heterogeneity in receptor expression between individual samples. Moreover, immunohistochemistry showed a stronger reduction in HER2 expression in lesions with higher vessel counts, the latter probably being responsible for better delivery of trastuzumab [18].

The efficacy of trastuzumab was further assessed and predicted through the correlation of molecular imaging biomarkers of apoptosis, glucose metabolism, and cell proliferation and tumor regression, in responsive and nonresponsive tumor-bearing cohorts, in two mouse models of breast cancer overexpressing HER2. In the first model, mammary tumors from mouse mammary tumor virus (MMTV)/HER2 transgenic female mice were transplanted into immunocompetent syngeneic FVB female mice. In the second model, nude athymic female mice were injected s.c. with human breast carcinoma cell lines. All mice were then treated with trastuzumab. Tumor glucose metabolism was assessed with ^18^F-FDG PET and cellular proliferation with ^18^F-FLT PET. Tumor cell apoptosis was assessed with an optical imaging tracer based on near-infrared (NIR) fluorescent 700-Annexin V; it will be discussed in the dedicated section. Animals were imaged weekly before and within 24 h of administration of trastuzumab, up to 3 weeks or until complete tumor regression was observed. The ^18^F-FLT PET imaging accurately predicted trastuzumab response in BT474 xenografts, but not in MMTV/HER2 tumors, which showed a moderate uptake even in regression. In both preclinical models, the uptake of ^18^F-FDG was not affected by trastuzumab treatment. Therefore, such imaging biomarkers were suitable for detecting early response and predicting treatment outcome, as well for evaluating new molecular targeted therapies in breast cancer [19].

The sensitivity of ^18^F-FLT in differentiating trastuzumab-sensitive and -resistant HER2 overexpressing xenografts has been assessed in a mouse xenograft model. Female athymic mice were implanted with trastuzumab-sensitive (BT474) or trastuzumab-resistant (HR6) cell lines. Mice were grouped into four cohorts: trastuzumab-treated BT474 and HR6 and the relative vehicle-treated control groups. The therapy included two treatments administered immediately after imaging at baseline and on day 3; the imaging acquisitions were repeated the day after each treatment. Longitudinal tumor volume was measured from T_2_-weighted MRI using a 7-T scanner, and mice were then injected i.v. with 293 ± 7.00 *μ*Ci of ^18^F-FLT for PET imaging. The tumor to muscle ratio (T : M) was used to compare ^18^F-FLT uptake in the tumors before and after treatment. The final results revealed the ability of ^18^F-FLT PET in distinguishing treated from untreated BT474-bearing mice. In contrast, because no differences were detected between treated and untreated HR6-bearing mice, these xenografts could be used to model clinical “nonresponders.” In this perspective, a significant difference in T : M was observed between trastuzumab-sensitive and -resistant cohorts after two treatments. Nonetheless, differences in tumor volume detected by MRI appeared as soon as ^18^F-FLT uptake differences [21].

In a similar study, the combined use of trastuzumab and rapamycin, a mammalian target of rapamycin (mTOR) inhibitor, was examined in inducing regression of HER2-positive mouse mammary tumors *in vivo*. The mTOR serine/threonine kinase complex (mTORC1) is a major effector in the phosphatidylinositol-3 kinase (PI3K) pathway, which is linked with trastuzumab resistance, making it a possible therapeutic target. Tumors from MMTV/HER2 transgenic female mice were transplanted in immunocompetent syngeneic wild-type FVB females, and mice were imaged for tumor cell death and glucose metabolism. The former was studied using NIR700-Annexin V, and the results are discussed in the dedicated section; the latter was studied using ^18^F-FDG PET. Treatment groups were constituted by vehicle (PBS), trastuzumab, rapamycin, or trastuzumab-rapamycin combination at the same dosages every other day. The combination treatment induced a decrease in ^18^F-FDG tumor uptake on day 7, which has been considered being linked to cell death, as confirmed by fluorescence imaging [22].

**Figure 1:**
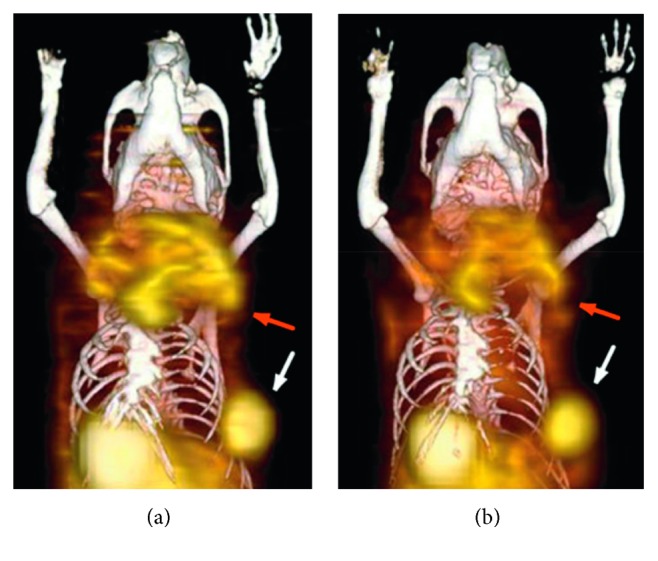
Female STAT1^–/–^ mice imaged with small-animal PET/CT using ^18^F-FES (a) and ^18^F-FFNP (b). Coronal 3-dimensional fused small-animal PET/CT images show a primary tumor in the left upper thoracic fat pad (red arrow) and a smaller tumor in the left lower thoracic fat pad (white arrow) (adapted from original research published in JNM. [25] © SNMMI).

Changes in the expression of tumor steroid hormone receptor after endocrine therapy might be used as predictors of treatment efficacy. In a preclinical model of human luminal breast cancer, intact female wild-type (129S6/SvEv) mice were injected s.c. with either SSM2 (spontaneous signal transducer and activator of transcription 1-deficient (STAT1^–/–^) mammary) or SSM3 tumor cell lines, derived from primary STAT1^–/–^ spontaneous tumors, into the right thoracic mammary fat pad. Small-animal PET/CT was performed using ^18^F-fluoroestradiol (^18^F-FES) for ER*α* imaging, ^18^F-fluoro furanyl norprogesterone (18F-FFNP) for PR imaging, and ^18^F-FDG for glucose uptake. Mice were injected in the tail vein with 11.1 MBq (300 *μ*Ci) of ^18^F-FDG, 11.1 MBq (300 *μ*Ci) of ^18^F-FFNP, or 5.55 MBq (150 *μ*Ci) of ^18^F-FES on separate imaging days. Moreover, mice underwent scans one hour after a radiotracer injection (Figure 1). Image analysis was performed based on T : M. Baseline radiotracer uptake confirmed previous immunohistochemical evaluations, with SSM3 tumors displaying the highest T : M for both ^18^F-FES and ^18^F-FFNP, and the SSM2, intermediate values. Hormonal treatment involved the administration of fulvestrant, a pure ER antagonist exhibiting competitive inhibition of receptor binding with estradiol, as well as proteasome-mediated degradation of ER. Control mice were treated with sunflower oil (vehicle). In SSM3, fulvestrant reduced uptake of both ^18^F-FES and ^18^F-FFNP, confirming reduced PR proteins levels and ER degradation; reduced ^18^F-FDG was detected as well. Tumor growth resulted interruption compared to control mice, confirming estrogen signaling inhibition. In SSM2 tumors, ^18^F-FFNP uptake resulted unexpectedly unchanged, whereas ^18^F-FES uptake was reduced as for SSM3. Thus, the growth of these tumors as well as their ^18^F-FDG uptake was unaffected; hence, such a cell line can be used to model “nonresponders.” Based on these results, ^18^F-FFNP PET can distinguish responders from nonresponders during hormonal therapy targeting ER*α*, with responders showing marked reduction in the uptake of this radiotracer. Moreover, such changes can be detected as early as three to four days after the initiation of fulvestrant therapy [25].

### 3.3. Potential Clinical Applications: PET

The major clinical applications of PET-CT include the detection and differentiation of primary breast lesions, lymph node staging, metastasis detection, and monitoring of the response to chemotherapy. Among radiotracers, ^18^F-FDG is the most commonly used, and it has been shown to be useful for monitoring the effects of chemotherapy and for identifying nonresponders to avoid ineffective chemotherapy. However, its limited sensitivity for small lesions makes ^18^F-FDG-PET unsuitable for the exclusion of early-stage disease [39–41].

Hence, more sensible and specific tracers for both diagnosis and therapy follow-up are needed. ^18^F-FBEM-Z_HER2:342_-Affibody seems to represent a noninvasive option for obtaining real-time information on changes in HER2 expression that facilitates patient selection for anti-HER2 therapy, such as 17-DMAG or trastuzumab treatment, and would result in optimal dose adjustment and treatment schedule for individual patients, as well as in the prediction of tumor response [16–18].

In the clinical field, ^18^F-FLT is not considered an ideal tracer for tumor detection and staging; however, it has been used as a marker for cellular proliferation. Thus, ^18^F-FLT- PET imaging could be used as an early biomarker of tumor response to therapy; it might be included among the techniques that can identify nonresponders earlier, and it might be considered an accurate predictor of long-term clinical outcomes [19, 21, 22, 42–45].

Finally, ^18^F-FFNP PET can distinguish responders from nonresponders during hormonal therapy targeting ER*α*; hence, it may represent a candidate for early stratification of patients receiving endocrine therapy [25]. Further preclinical as well as clinical trials would be needed to confirm the aforementioned hypotheses on both therapeutic and diagnostic strategies.

## 4. Magnetic Resonance Imaging

Magnetic resonance imaging (MRI) is an advanced clinical and research technique for morphological, structural, and functional imaging. It allows noninvasive evaluation of soft tissues in multiple planes (both two- and three-dimensional imaging) [46, 47]. The physical principle of MRI is the magnetic field, the so-called magnetic moment, of the hydrogen proton, which is present in a very large amount within the body in the form of water. Such a magnetic moment can be manipulated and deflected, longitudinal and transversal magnetization, with external magnetic fields and radiofrequency pulses, and the return to the equilibrium state produces a signal recorded and converted to an image by the MR system [48]. The contrast in the final image depends on the intrinsic chemical structure of the tissues imaged (i.e., proton density) and on the recovery time of magnetization of such protons. The latter, obtained with the application of a frequency pulse, induce changes in proton spin. The recovery time to get the initial state is known as T_1_ and T_2_ relaxation. T_1_ relaxation, or spin-lattice relaxation, depends on the interaction of nuclei with external surroundings; it is the time, in milliseconds, required to recover 63% of the longitudinal magnetization. T_2_ relaxation, or spin-spin relaxation, is produced by random interactions with similar nuclei; it is the time, in milliseconds, required to reduce the transverse magnetization to 37% of its initial value [48]. Based on weighting on T_1_ and T_2_ relaxation, many sequences can be developed, aiming to reveal both evident and subtle structural and physiopathological changes.

### 4.1. Dynamic Contrast-Enhanced Imaging

Dynamic contrast-enhanced (DCE) imaging is a perfusion MRI application, based on T_1_-weighted (T_1_-w) acquisition, for the assessment of microcirculation and, eventually, antiangiogenic treatment responses [49]. The T_1_-w sequence used is characterized by high temporal resolution, and it is dynamically performed before, during, and after an intravenous injection of gadolinium-based contrast agents. Such agents reduce the T_1_ of tissues, which is captured by the scanner as an increased signal intensity. Postprocessing allows extracting semiquantitative and quantitative parameters, which reflect the tumor's vascularization status and permeability [50–52]. Semiquantitative parameters are maximal contrast enhancement (Cpeak, % base), time to peak (TTP, 289 s), speed of contrast uptake (wash-in, % base/min), and clearance rate of the contrast material (wash-290 out, % base/min) [53]. These parameters are either automatically obtained with acquisition software by placing a region of interest (ROI) on the lesion or through in-house scripts developed for processing software such as BioMAP (Novartis, Basel, Switzerland), MATLAB (The MathWorks Inc., Massachusetts, USA), or ImageJ (National Institutes of Health, NIH, USA) [53–55].

Quantitative measurements, derived from the analysis of multicompartmental pharmacokinetic models, estimate contrast kinetic parameters, such as *K*^trans^ (volume transfer constant); *v*_*e*_ (volume fraction of extracellular, extravascular space, EES); and *K*_*ep*_ (exchange rate constant), which is the *K*^trans^/*v*_*e*_ ratio. The most used compartmental pharmacokinetic models are the generalized kinetic model, also called the Tofts model, Brix model, and shutter-speed model. In particular, the first requires the quantification of the contrast agent concentration by arterial input function (AIF), which is subsequently used in the Tofts mathematical model to calculate the aforementioned quantitative parameters. There are different ways of estimating AIF; for instance, direct sampling of AIF is possible with arterial blood sampling, but it is considered invasive for patients and very challenging in small rodents. Another way is AIF estimation based on MR images (image-derived AIF), which is noninvasive, but it is time consuming in postprocessing; it can only be performed by placing ROIs on large vessels, such as the aorta, and higher doses of contrast agents are required [56]. The AIF can also be calculated by evaluating the contrast agent concentration in reference tissues; for instance, by placing an ROI on thigh muscles, whose perfusion rate, extraction fraction, and extracellular volume are known [57]. However, the most common method remains the population-based AIF drawn from scientific literature [56]. The Brix model estimates the kinetic parameters directly from relative signal intensity curves, which allows reducing errors, particularly in murine models, from image-derived AIF. Due to its physicochemical properties, contrast agents fail to enter the cells, but act only in the EES. However, both Tofts and Brix models assume that the water flux through cells is so rapid that the contrast agent acts on all water protons. In contrast, the shutter-speed model rejects this hypothesis [58, 59]. Indeed, it uses a new parameter, *τ*i, that evaluates how long water protons rest inside the cell, thus quantifying the MR effects on longitudinal magnetization (for all mathematical functions, refer to [59]).

### 4.2. Diffusion Weighted Imaging

Diffusion is the property of molecules to move inside a system, in relation to physicochemical properties of the surroundings. Free water molecules move without a preferential direction (i.e., the Brownian motion). Diffusion-weighted imaging (DWI) is an MRI approach to detect water's random movements inside tissues. When pathological insults change the tissues structure and their biological characteristics, DWI sequences can detect such changes and give significant and early diagnostic indications, particularly in oncology but also in vascular pathologies (e.g., stroke) [60]. The “factor b” or b-value depends on the timing and spacing of the gradients used to generate diffusion images. To obtain valid DW acquisitions, multiple b-values are used, and depending on their higher or lower values, different information can be obtained [61–63]. DWI acquisitions can be elaborated with mathematical algorithms to obtain parametric maps, such as apparent diffusion coefficient (ADC) maps; the ADC map allows determining the reduction of water molecule diffusion caused by cell membranes, thus estimating the cellularity of tissues. For example, the ADC map shows lower intensity in tumor tissues, characterized by a higher cellularity, than in normal tissues [51]. Various mathematical models have been developed to highlight the different properties of diffusion of water molecules when they are inside a complex system, such as human tissues. Currently, the most used way of calculating ADC maps is the Gaussian mono-exponential mathematical model, which is based on the hypothesis that water molecules move freely between body tissues and their displacement follows a Gaussian distribution. However, it is known that the mono-exponential model is not appropriate for evaluating ADC in many tissues [64]. Consequently, different models have been used, such as the intravoxel incoherent motion model (IVIM) and non-Gaussian compartmentalized and noncompartmentalized models, to evaluate ADC as well as the features of other tissues. The IVIM model, using low b-values (i.e., 0–50 s/mm^2^), can include the contribution of microvasculature to the image signal. Compartmentalized models divide voxels in compartments to evaluate the features of the main tumor tissue (i.e., intracellular, interstitial, and intravascular water). In contrast, noncompartmentalized models, such as kurtosis and stretched exponential model, include in the model parameters the possible compartments without assuming a specific number and consider both spatial heterogeneity and temporal heterogeneity (i.e., the temporal and spatial displacement of water molecules in a given tissue) [63].

### 4.3. Therapy Response and Detection of Responders vs. Nonresponders

**Figure 2:**
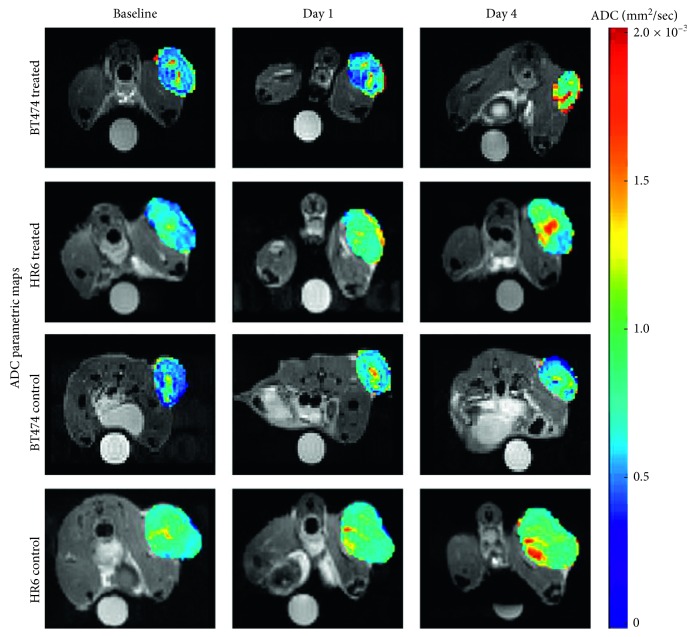
Diffusion-weighted (DW) magnetic resonance imaging (MRI). Apparent diffusion coefficient (ADC) parametric maps of a representative mouse from each cohort. The columns indicate baseline, day 1, and day 4 posttreatment, whereas each row indicates each of the four experimental groups. Regions with noticeably increased ADC values are observed within the center of the treated and control HR6 cohorts (reprinted from [20], copyright with permission from © 2014 Neoplasia Press, Inc., published by Elsevier Inc.).

**Figure 3:**
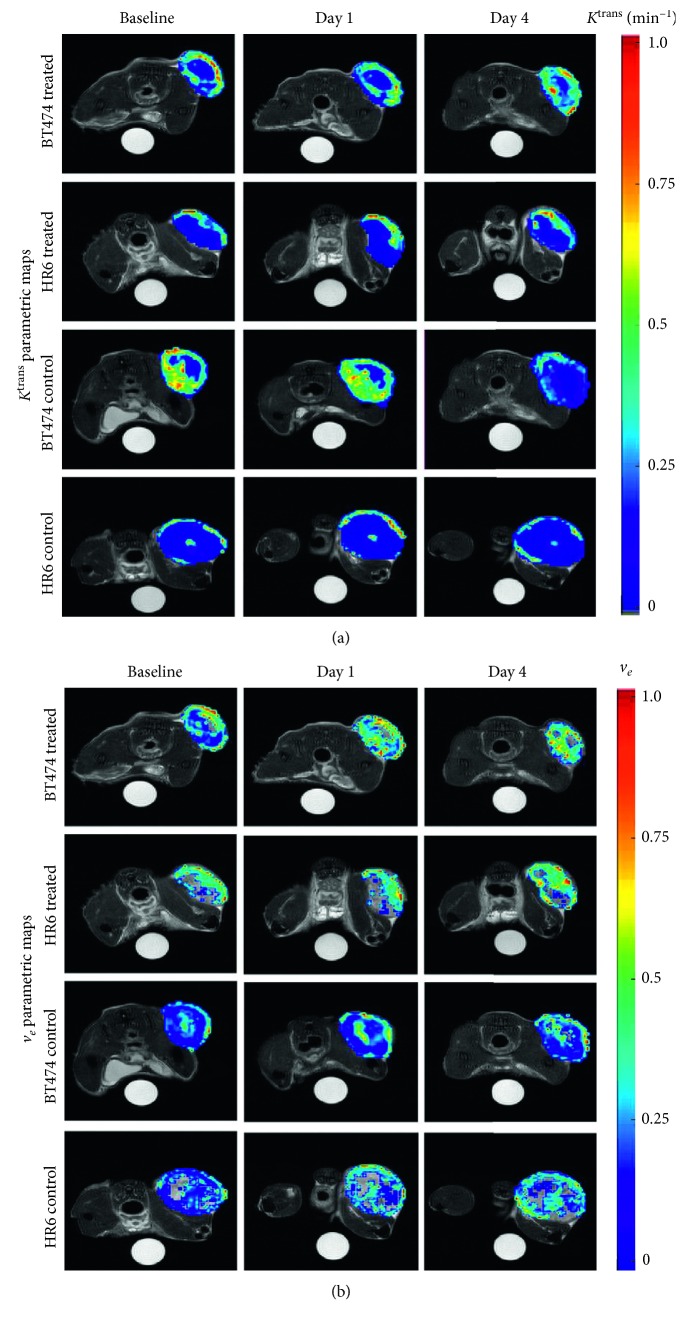
Dynamic contrast-enhanced (DCE) magnetic resonance imaging (MRI) parametric maps. In (a) *K*^trans^ and in (b) *v*_*e*_ of a representative mouse from each group. The *K*^trans^ parametric maps reveal enhancement along the periphery with increasing trends in the BT474-treated group. The *K*^trans^ parametric maps remain fairly consistent in HR6-treated groups, while the BT474 and HR6 control groups slightly decrease over time. The *v*_*e*_ parametric maps reveal variations within all the observed tumors, with increased levels in the treated groups compared to the control groups (reprinted from [20], copyright with permission from © 2014 Neoplasia Press, Inc., published by Elsevier Inc.).

Using the experimental design described in the PET chapter, DW and DCE-MRI sequences were tested for their capacity in measuring the antiproliferative and antivascular effects of trastuzumab and for their sensitivity in identifying responsiveness in HER2+ breast cancer xenograft models. Briefly, female athymic mice were implanted with trastuzumab-sensitive (BT474) or trastuzumab-resistant (HR6) cell lines. Mice were grouped into four cohorts: trastuzumab-treated BT474 and HR6 and the relative vehicle-treated controls. The therapy included two treatments administered immediately after imaging at baseline and on day 3; the imaging acquisitions were repeated the day after each treatment. Tumor volume was measured from T_2_-weighted images; DW images were acquired using a standard pulsed gradient spin-echo sequence, and DCE T_1_-weighted images were acquired using a spoiled gradient echo sequence with an i.v. bolus of 0.05 mmol/kg Gd-DTPA. Differences in tumor volume were not detectable until the last imaging session, when smaller tumor volumes were revealed in BT474-treated compared to the control group, in HR6-treated compared to the control group, and in BT474-treated compared to the HR6-treated group. In summary, changes in the ADC (Figure 2) and *K*^trans^ (Figure 3(a)) allowed the differentiation between responders and nonresponders late in the therapeutic protocol, whereas the *v*_*e*_ of DCE was more sensitive in the early detection of responsiveness (Figure 3(b)), revealing it before tumor size changes [20].

### 4.4. Potential Clinical Applications: MRI

The MRI is the most sensitive imaging modality for detecting breast cancer in clinical settings. In this field, MRI is indicated for screening because it is able to detect breast cancer when it is still occult clinically, mammographically, and ultrasonographically. In addition, breast MRI is used to monitor the response to neoadjuvant treatments and to evaluate the integrity of the implants [65]. The DCE and DWI are additional MRI techniques with a strong potential for reducing false-positive diagnosis and unnecessary biopsies. Such sequences improve early assessment, monitoring, and prediction of tumor response to therapy and allow the evaluation of residual tumors [66, 67].

The preclinical study discussed in this manuscript highlighted the ability of DCE and DWI to determine the antiproliferative and antivascular effects of trastuzumab in HER2+ breast cancer xenografts. The results revealed that changes in ADC and *K*^trans^ could distinguish responders from nonresponders, albeit late, whereas the *v*_*e*_ of DCE showed a timely detection of responsiveness to a therapeutic protocol [20]. Thus, such sequences might be directly applied in clinical trials to confirm the reported results.

## 5. High-Frequency Ultrasonography (HFUS)

High-frequency ultrasonography (HFUS) is a noninvasive, cost-effective imaging technique, which can provide real-time images with high spatial resolution [24]. It is based, just as traditional ultrasonography, on the piezoelectric effect of some natural elements, such as quartz, that generate ultrasound (US) wave trains. Such ultrasonic waves travel through soft tissue and they are, in part or fully, reflected back. The distance that the US wave has to cover back and forth, as well as the amount of US wave reflected, are detected and processed by dedicated systems to reconstruct a grayscale, two-dimensional image (brightness or B-mode). The physical-chemical structure of soft tissues encountered by the US wave, in particular their acoustic impedance, determine their US features (i.e., echogenicity), thus allowing fine structural evaluation of soft tissues. In contrast, bones, as well as air, do not allow further transmission of US; the former because it absorbs all US, the latter because it reflects them all. Thanks to technological advancements, HFUS now allows studying small laboratory animals with excellent spatial resolution, but by sacrificing the depth of penetration (which is inversely proportional to US waves' frequency). All clinical applications are available in preclinical US systems, such as motion (M-) mode and tissue-Doppler for cardiologic application and spectral-, power-, and color-Doppler for vascular evaluation [68]. Moreover, US probes' motorization allows for three-dimensional acquisitions [69]. In clinical applications, probes' frequencies are in the range of 2 and 15 MHz. In contrast, small imaging studies adopt probes with frequencies of 20 MHz up to 70 MHz, for the analysis of superficial structures, when very high spatial resolution is needed [70]. The HFUS provides morphologic images of organs and lesions, and it allows longitudinal monitoring of treatment response, in terms of factors such as cytoreduction and vascularization changes.

**Figure 4:**
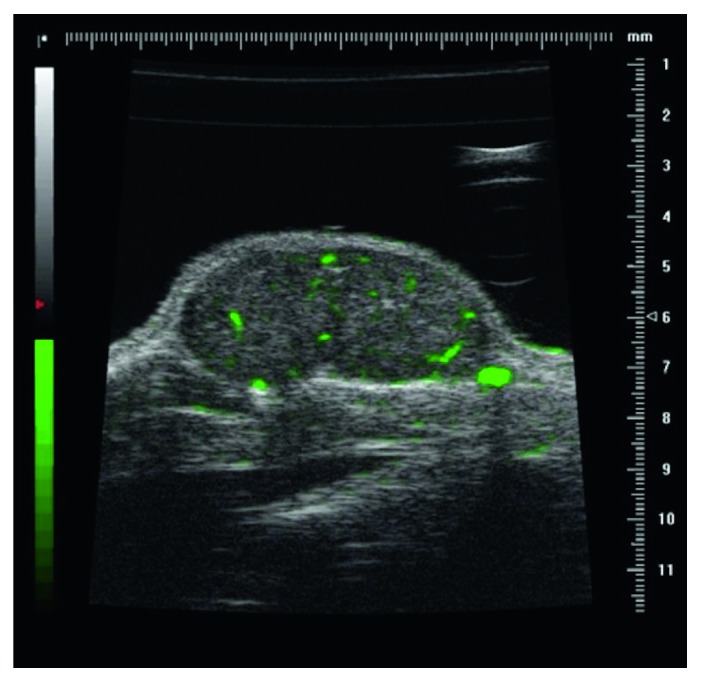
In vivo contrast-enhanced high-frequency ultrasonography with antivascular endothelial growth factor receptor 2 (VEGFR2) labeled microbubbles in a xenograft mouse model of breast cancer. The green bar on the left is a colorimetric scale for the specific ultrasonographic contrast agent signal intensity (courtesy of Mancini M., Greco A., unpublished).

Microbubbles (MBs) contrast-enhanced ultrasonography (CEUS) improves the visualization and increases the physio-anatomical information on tumor vascularity and angiogenesis. The enhancing effect produced by MBs occurs thanks to their gaseous nuclei, which reflect most of the US wave, resulting in hyperechogenicity, thus causing a very high contrast compared to the tissues' background [71]. In particular, MB-based ultrasonographic contrast agents (UCAs) have been developed to specifically target tumor vasculature via conjugated peptides and antibodies. Among these, the vascular endothelial growth factor receptor 2 (VEGFR2) has been used as a target for UCAs [35, 72, 73] because it plays an important role as a regulator of angiogenesis in tumor vasculature (Figure 4).

### 5.1. Tumor Response

Many therapeutic agents have been developed to inhibit the functions of the VEGFR2 receptor. Thus, the use of anti-VEGFR2 UCAs would allow not only the detection of this receptor in tumors, but also its quantification in longitudinal follow-up as a measure of response to therapy. To investigate a mouse model of murine breast cancer, MBs conjugated to anti-VEGFR2 were injected into the tail vein. The UCA was allowed to circulate for 4 minutes, a time sufficient for the binding of targeted MBs and the washout of the free circulating ones. Mice were then evaluated *in vivo* with a HFUS system, with the acquisition of two sets of images, before and after the application of a high-power ultrasonic destruction sequence (20 cycles, 10 MHz, mechanical index of 0.59). The difference in video intensity between the pre- and post-destruction images was measured, providing a semiquantitative measure of the retention level of the UCA in the tumor. The retention of anti-VEGFR2 MBs, measured as explained, was significantly higher compared to the nontargeted UCA. These results validated the use of molecular ultrasonography for *in vivo* detection and quantification of VEGFR2 expression in breast cancer models and for the evaluation and longitudinal monitoring of new antiangiogenic drug efficacy [24].

### 5.2. Potential Clinical Applications: HFUS

Ultrasonography is already among the first-line imaging modalities in the clinical setting for many organs, such as the mammary gland, ovaries, and pancreas, for the early detection, molecular profiling, angiogenesis level evaluation, and monitoring of tumors [74–76]. From a translational perspective, HFUS devices have been used successfully for clinical applications, e.g., to study the anterior segments of the eye and the skin [77]. Multiple preclinical studies have shown that US molecular imaging is a versatile, safe, and accurate tool for the evaluation of therapies and for theranostic applications. Indeed, US imaging in combination with MBs may allow better assessment of treatment regimens and the differentiation of responders from nonresponders, and it may also be useful for minimizing drug doses in a treatment protocol [75, 76]. The translation of the CEUS imaging approaches into clinical applications may be easily achievable, since ultrasonography and CEUS are already available and often used in the clinical field [78]. There are several clinical applications of this method, including the assessment of inflammation, such as in inflammatory bowel disease, or transient myocardial ischemia and atherosclerosis.

Among the UCAs, BR55 was the first targeted VEGFR2 contrast agent introduced as a clinical grade. The BR55 is a gas core of a mixture of perfluorobutane and nitrogen used to visualize the expression levels of the molecular marker VEGFR2 to evaluate angiogenesis in various tumor types including breast cancer. Following extensive validation in various preclinical animal models, BR55 was successfully used to monitor the effects of antiangiogenic drugs. Further, targeted UCAs should be developed and tested to refine clinical applications of ultrasonography and to support the development of novel chemotherapeutic agents [35, 76].

## 6. Optical Imaging

Optical Imaging includes various preclinical imaging techniques based on the detection of light, i.e., photons, at different wavelengths produced by bioluminescence, i.e., light-emitting molecules oxidized by luciferases; fluorescence, i.e., fluorophores excited by laser beam; and photoacoustic, i.e., excitation of either endogenous or exogenous molecules by a laser beam [79]. The choice of the technique depends on the processes in study, e.g., bioluminescence is usually adopted as a surrogate for tumor growth [80]. In contrast, fluorescence is used to study biodistribution as well as physiologic and pathologic processes at the cellular and molecular level [81]. Finally, the photoacoustic effect is best used for the evaluation of microcirculation and hemoglobin oxidation status in tissues [82].

**Figure 5:**
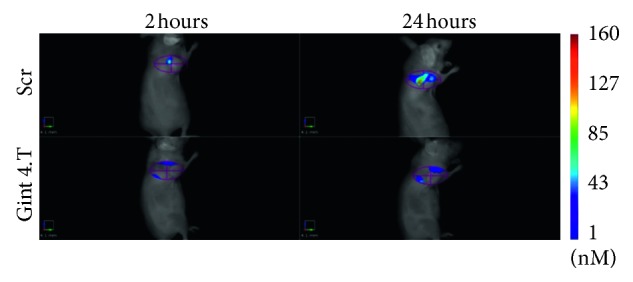
In vivo fluorescence molecular imaging of breast cancer (MDA-MB-231) xenografts. Tracking of bone marrow mesenchymal stem cells labeled with a NIR fluorophore, pretreated with a nuclease-resistant aptamer (Gint4.T) or scrambled aptamer (Scr) (reprinted from [32], copyright with permission from CC by NC 4.0).

In the context of this review, among the other optical imaging techniques, only fluorescence imaging has been considered. For in-depth preclinical bioluminescence and photoacoustic applications, readers might refer to other recently published reviews [83–85]. As mentioned earlier, the use of fluorescence imaging allows visualizing, noninvasively and without the use of ionizing radiations, and biological processes at the molecular level (Figure 5; [32]), including those influencing tumor behavior and response to drugs [23, 32].

Fluorescence imaging can be performed either in two dimensions (fluorescence reflectance imaging, FRI) or in three dimensions (fluorescence molecular tomography, FMT). Both the acquisition modes are based on the ability of a particular exogenous substance, known as fluorophores, to emit light of well-known wavelength once excited by a laser beam of proper wavelength. A digital charge-coupled device camera detects such light, and it transmits the signal to a workstation for image processing. The main difference between FRI and FMT is depth of penetration, with the former allowing only for superficial fluorescence detection and the latter allowing for high-sensitivity quantification of the molecules studied [86]. Moreover, to overcome the low penetration depth of fluorescence in the visible wavelength, current devices work in the NIR wavelength, i.e., usually between 600 and 900 nm for the emission spectrum [87].

Quantum dots (QDs) are semiconductor nanoparticles that work as traditional fluorophores, but with far greater photostability and brightness. Moreover, their excitation can lead to emission of different “colors,” i.e., different wavelengths. As for other nanoparticles, QDs can be decorated for targeted imaging, thus enhancing their specificity, or for lengthening their circulation half-life [88, 89].

### 6.1. Diagnosis and Tumor Response

As described before, the NIR700-Annexin V optical imaging probe has been demonstrated as a molecular biomarker of tumor cell apoptosis in two studies. In both studies, this approach was helpful, in association with PET imaging, for detecting early response and predicting treatment outcome [19, 22].

In particular, mammary tumors from MMTV/HER2 transgenic female mice were transplanted into immunocompetent syngeneic FVB, and nude athymic female mice were injected s.c. with human breast carcinoma cell lines and then treated with trastuzumab. Animals were imaged weekly before and within 24 h of administration of trastuzumab, up to 3 weeks or until complete tumor regression was observed. The results suggested that molecular imaging of apoptosis might accurately predict trastuzumab-induced regression of both MMTV/HER2 transgenic mouse mammary tumors and BT474 xenografts, as witnessed by the higher accumulation of NIR700-Annexin V in the tumor xenografts [19].

In a similar study, tumors from MMTV/HER2 transgenic female mice were transplanted in immunocompetent syngeneic wild-type FVB females, and mice were imaged for tumor cell death, with NIR700-Annexin V. As described above, groups were treated with vehicle (PBS), trastuzumab, rapamycin, or trastuzumab-rapamycin combination every other day. The results showed that single-agent treatments did not alter tumor NIR700-Annexin V uptake compared to vehicle treatment, but their combination significantly increased absolute tumor fluorescence: hence, demonstrating an early induction of tumor cell death [22].

The NIR-QDs have been developed and have become advanced preclinical contrast agents for efficient tumor imaging [23]. Generally, nanoparticles can be transported and accumulated in the tumor through passive and/or active mechanisms. In passive targeting, nanoparticles accumulate in the tumor through the enhanced permeability and retention effect, which is linked to the structural peculiarities of tumor tissue and is widely used by most anticancer drugs. The active targeting takes advantage of peculiar receptors expressed by neoplastic tissues, to which the targeting moieties bind specifically. Fluorescent QDs were evaluated in a HER2/neu-positive breast cancer model using both passive and active targeting. For passive tumor targeting, 705 nontargeted QDs coated with polyethylene glycol (PEG) were used as contrast agents, whereas 705 ITK carboxyl QD were bound to anti-HER2/neu 4D5scFv antibodies (QD-4D5scFv) for active tumor targeting. *In vivo* whole-body fluorescence imaging was used to analyze the accumulation of the probes (QD-PEG and QD-4D5scFv) at the tumor site. The maximum difference between QD-PEG and QD-4D5scFv signals was registered 3 hours after i.v. injection, with a 1.5-fold increase. Overall, these data have shown that both passive and active deliveries allow successful imaging of tumors, but QD-4D5scFv fluorescent signal was considerably stronger than that of QD-PEG. Therefore, the choice of passive or active targeting strategy depends on the objectives of the study. Passive tumor targeting was the method of choice to anatomically identify the malignant process. However, the advantage of active tumor targeting is in the ability to analyze both the location of the tumor and its molecular profile. The molecular characteristics might then be used in selecting the right antineoplastic agents and, eventually, in correcting the planned therapeutic strategy [23].

### 6.2. Potential Clinical Applications: Optical Imaging

Optical imaging includes very sensitive, easy to manage, and relatively cost-effective modalities, with short acquisition times that allow the visualization of physio-pathological processes *in vivo* with high specificity and in real time [90]. The disadvantages derive from the diffusion, the absorption, and the wavelength of light used, which influence the image resolution and the depth of penetration in the tissues, and consequently the ability to obtain quantitative data [91]. Penetration depth is not a crucial aspect in mice due to their small size, and indeed optical imaging techniques are already suitable for preclinical research. However, optical imaging cannot yet be translated into clinical practice, but the molecular markers discovered with such techniques might be translated [91]. In preclinical research, these modalities have been applied to monitor gene expression and to study toxicology, viral infection, tumor growth, and metastases in real time. In particular, the ability to visualize and quantify blood vessel development in metastases, or to evaluate HER2 expression *in vivo* and monitor therapy response, makes optical imaging a promising tool to study angiogenesis and carcinogenesis and in choosing effective treatments as an alternative to nuclear medicine techniques [92–94]. The potential clinical translation of fluorescence imaging might be directly possible when penetration depth is not an issue, for example, in endoscopic setup, for intraoperative assessment of surgical margins in specific organs and for superficial breast imaging.

The NIR optical imaging is not yet approved for routine clinical use; however, some studies have revealed its ability to distinguish benign from malignant breast lesions in humans, with the use of NIR optical spectroscopy alone and in combination with MRI [95, 96]. The NIR-QDs can be visualized in deep tissues, and this feature may be suitable for the guided administration of chemotherapeutic agents for the evaluation of micrometastasis sites and for performing an adequate tumor resection in surgery [97, 98]. The QDs have been used in many animal models for molecular imaging of cancer with different targets. However, the main drawback in their clinical translatability is the toxicity of their cadmium core. Hence, a paramount step to enable clinical translation is to determine QD toxicity and reduce their doses and the development of a new generation of cadmium-free QDs [99].

## 7. Improving Molecular Imaging Clinical Translatability

*In vivo* molecular imaging still has great potential to contribute to biomedical research, particularly in the preclinical setting, but it is also becoming a useful tool for translational research, helping to understand several features at the molecular, cellular, and organic level. Usually, for clinical translation, a key role of *in vivo* imaging is drug development: understanding drug mechanism of action, possible patient stratification into responders and nonresponders, and to predict the effectiveness of therapy or to recognize early resistance [100]. Various studies with the techniques mentioned in this manuscript have to be performed in clinical trials, before they can be reliably used for patient care, particularly in the oncology field. For example, DCE-MRI enables the evaluation of tumor neo-vascularization, which is useful for early identification of treatment failures, allowing rapid implementation of second-line therapy [101]. In addition, conventional breast DCE-MRI has been demonstrated in clinical studies to enable early identification of tumor response in patients with breast cancer undergoing neoadjuvant chemotherapy (NAC). The accurate assessment of the response to NAC treatment before surgery offers the possibility of avoiding unnecessary, mutilating procedures [102, 103]. Implementing the use of PET in preliminary breast cancer studies may support clinical decision-making through monitoring receptor expression during treatment, aiming at developing personalized treatment strategies and/or predicting prognosis [104]. Interesting results, for example, were collected on sensitivity and specificity of PET in identifying NAC responders in TNBC mouse models [105]. The translation of novel radiotracers for precision imaging would improve the clinical management and outcome of patients affected, in particular TNBC patients, who rely upon a limited number of therapeutic opportunities with poor prognosis [105]. Furthermore, CEUS using targeted VEGFR2 MBs has been evaluated in clinical settings, and is considered as an additional screening modality, other than mammography and conventional ultrasonography, to improve diagnostic accuracy for early detection of breast cancer or even for its precursor lesions. Such an approach, with the other cited imaging technologies, may improve the ability to visualize the molecular characteristics of breast cancer in each patient, with high sensitivity and specificity, improving all the phases of patient management [73].

## 8. Conclusions and Future Perspectives

Personalized medicine is still in a developing stage. The customization of therapies represents a desirable future, in which diseases are detected earlier and treatments are tailored to the profile of individual patients. Preclinical molecular imaging may be one of the keys for rapid advancement in this field. Its ability to characterize molecular features of different histotypes and to discriminate responders from nonresponders could empower the translational utility of mouse models. From this perspective, the use of PDX in testing therapeutic responses would represent a real-time personalization for individual patients, even if such models may lack recapitulation of the human tumor microenvironment, as well as immune response. Their disadvantages could be related to long bureaucratic times, expensive costs, ethical considerations, and experimental failures, but animal models remain an irreplaceable tool to improve and better comprehend molecular mechanisms and patterns of oncologic diseases.

## Figures and Tables

**Table 1:** Summary of the molecular preclinical imaging techniques used with regard to personalized medicine. The columns define the techniques, tracers, and cell lines used to model human breast cancer, specific receptor targets, and therapies.

Imaging	Tracer	Cell line	Receptor	Treatment	Reference
PET	^18^F-FBEM-Z_HER2:342_	MDA-MB-361	HER2	17-DMAG	[16, 17]
		MCF7			
		MDA-MB-468			
		MCF7/clone18			
		BT474			
PET	^18^F-FBEM-HER_2:342_	BT474	HER2	Trastuzumab	[18]
NIR PET	700 Annexin-V	BT474-AZ MMTV/HER2	HER2	Trastuzumab	[19]
	18F-FDG				
	18F-FLT				
MRI (DW–DCE)		BT474	HER2	Trastuzumab	[20]
		HR6			
PET–MRI	^18^F-FLT	BT474	HER2	Trastuzumab	[21]
		HR6			
NIR PET	700 Annexin-V	MMTV/HER2+	HER2	Trastuzumab Rapamycin	[22]
	^18^F-FDG				
NIR	QD-PEG	SKBR-3	HER2		[23]
	QD-4D5scFv				
HFUS	Anti-VEGFR2-MBs	67NR	VEGFR2		[24]
PET	^18^F-FES	SSM2	ER*α*–PR	Fulvestrant	[25]
	^18^F-FFNP	SSM3			

## Abbreviations

(17-DMAG): 17-(dimethylaminoethylamino)-17-demethoxygeldanamycin
^18^F-FBEM-HER_2_: _342_:^18^F-fluorobenzamidoethylmaleimide
^18^F-FBEM-Z_HER2_: _342_:N-2-4-^18^F-fluorobenzamidoethylmaleimide
^18^F-FDG: 2-deoxy-2-^18^F-fluoro-d-glucose
^18^F-FES: ^18^F-fluoroestradiol
^18^F-FFNP: ^18^F-fluoro furanyl norprogesterone
^18^F-FLT: ^18^F-fluoro-3-deoxythymidine
^18^F-FMISO: ^18^F-fluoro-misonidazole
ADC: Apparent diffusion coefficient
AIF: Arterial input function
CEUS: Contrast-enhanced ultrasonography
Cpeak: Maximal contrast enhancement
DCE: Dynamic contrast-enhanced
DW: Diffusion-weighted
EES: Extracellular, extravascular space
ER*α*: Estrogen receptor-*α*
ErbB2: Avian erythroblastosis oncogene B
FRI: Fluorescence reflectance imaging
FMT: Fluorescence molecular tomography
HFUS: High-frequency ultrasound
HER2: Human epidermal growth factor receptor 2
Hsp 90: Heat shock protein 90
i.p: Intraperitoneal
i.v.: Intravenous
IVIM: Intravoxel incoherent motion model
*K*_*ep*_: Exchange rate constant
*K*^trans^: Volume transfer constant
MBs: Microbubbles
MMTV: Mouse mammary tumor virus
MRI: Magnetic resonance imaging
mTOR: Mammalian target of rapamycin
mTORC1: Serine/threonine kinase complex
NAC: Neoadjuvant chemotherapy
NIR: Near-infrared
PBS: Phosphate buffered saline
PDX: Patient-derived tumor xenograft
PEG: Polyethyleneglycol
PET: Positron emission tomography
PI3K: Phosphatidylinositol-3 kinase
PR: Progesterone receptor
s.c.: Subcutaneously
QDs: quantum dots
ROI: Region of interest
SSM2: Spontaneous signal STAT1^–/–^ mammary
STAT1^–/–^: Spontaneous signal transducer and activator of transcription 1-deficient
TNBC: Triple negative breast cancer
TTP: Time to peak
UCAs: Ultrasonographic contrast agents
ve: Volume fraction of EES
VEGFR2: Vascular endothelial growth factor receptor 2.
